# Comparison of clinical characteristics and treatment outcomes between initially diagnosed type 1 and type 2 diabetes mellitus patients presenting with diabetic ketoacidosis

**DOI:** 10.1186/s12902-024-01649-7

**Published:** 2024-07-15

**Authors:** Ornwimol Mookpaksacharoen, Sawaraj Choksakunwong, Raweewan Lertwattanarak

**Affiliations:** 1 Division of Endocrinology and Metabolism, Department of Medicine, Faculty of Medicine Siriraj Hospital, Mahidol University, 2 Wanglang Road, Bangkoknoi, Bangkok, 10700 Thailand; 2 Diabetes, Thyroid and Endocrine Clinic, Siriraj Piyamaharajkarun Hospital, Faculty of Medicine Siriraj Hospital, Mahidol University, Bangkok, Thailand

**Keywords:** Diabetic ketoacidosis, Newly diagnosed diabetes, Type 1 diabetes, Type 2 diabetes

## Abstract

**Objective:**

Patients with type 1 diabetes (T1DM) and type 2 diabetes (T2DM) can present with diabetic ketoacidosis (DKA) as the first manifestation. Differentiating types of newly diagnosed diabetes could provide appropriate long-term management. Therefore, we conducted this study to compare clinical characteristics and outcomes between initially diagnosed type 1 and type 2 diabetes mellitus patients presenting with DKA.

**Materials and methods:**

A retrospective study was conducted on adult patients who presented with DKA as the first diagnosis of diabetes in our tertiary hospital between January 2005 and December 2019. Demographic data, precipitating causes, laboratory investigations, treatment, and outcomes were obtained by chart review. The primary outcome was to compare the clinical characteristics of initially diagnosed patients with T1DM and T2DM who presented with DKA.

**Results:**

A total of 100 initially diagnosed diabetic patients who presented with DKA were analyzed (85 T2DM patients and 15 T1DM patients). Patients with T1DM were younger than patients with T2DM (mean age 33 ± 16.2 vs. 51 ± 14.5 years, *p* value < 0.001). Patients with T2DM had a higher body mass index, family history of diabetes, precipitating factors, plasma glucose, and lower renal function than those with T1DM. There was no difference in resolution time or DKA management between T1DM and T2DM patients. The overall mortality rate of DKA was 4%.

**Conclusion:**

In this population, most adult patients who presented with DKA had T2DM. Older age, obesity, a family history of diabetes, and the presence of precipitating factors were strong predictors of T2DM. We can implement the same clinical management for DKA in both T1DM and T2DM patients. However, T2DM patients had longer hospitalization than T1DM patients. After DKA resolution for 12 months, more than half of patients with T2DM could discontinue insulin. Therefore, the accurate classification of the type of diabetes leads to appropriate treatment.

**Supplementary Information:**

The online version contains supplementary material available at 10.1186/s12902-024-01649-7.

## Introduction

Diabetes mellitus is one of the most common noncommunicable diseases worldwide [1]. According to global data in 2021, 537 million adults had diabetes [2]. A previous study in Thailand [3] reported an increase in the prevalence of diabetes over the 10-year period from 7.1 to 8.9% in men and 8.3–10.8% in women (2004–2014). Many diabetic patients are asymptomatic or have mild hyperglycemic symptoms. However, some patients can present with diabetic ketoacidosis (DKA) as the first manifestation [4]. According to the 2019 World Health Organization (WHO) classification of diabetes mellitus [5], there are six types of diabetes that are classified by pathophysiology, clinical characteristics, laboratory investigations, and long-term management. Type 1 diabetes mellitus (T1DM) results from autoimmune destruction of the beta cells of the pancreas, causing an insulinopenic state in most patients [6]. At diagnosis, between 70 and 90% of children with T1DM have evidence of an immune-mediated process with islet-cell autoantibodies against glutamic acid decarboxylase 65 (GAD), islet antigen 2 (IA2), or insulin autoantibodies (IAA) [7]. T1DM patients usually have a normal or low body mass index (BMI) [8]. According to data from the DIAMOND project initiated by the WHO in 1990 [9], the highest incidence of T1DM was among children 10–14 years old. However, in a recent study of 2,844 Thai diabetic patients diagnosed before the age of 30 years [10], the proportion of type 1 diabetes was only 68.05%, 56.97%, 41.96%, and 27.75% among patients with ages of onset from 10 to < 15, 15 to < 20, 20 to < 25, and 25 to < 30 years, respectively. All patients with T1DM need lifelong insulin therapy [6].

Type 2 diabetes mellitus (T2DM) is the most common type of diabetes mellitus in adults and results from insulin resistance and an insulin secretory defect due to beta cell dysfunction [2]. T2DM patients are characterized by symptoms and signs of insulin resistance (such as obesity and acanthosis nigricans) [11] and a family history of diabetes mellitus [12].

DKA is a life-threatening condition resulting from insulin deficiency and the rapidly increasing counterregulatory hormones that cause hyperglycemia with ketoacidosis [13]. From a recent systematic review [14], the range of DKA prevalence among T1DM adult patients was 0-128 per 1000 people. From a multicenter study in European countries [15], 17.4% of T1DM patients and 0.5% of T2DM patients had DKA at the time of diagnosis with diabetes. According to the data from 2,844 Thai diabetic patients diagnosed before the age of 30 years [10, 16], 67.8% of T1DM patients and 12% of T2DM patients presented with DKA at diagnosis, and the annual incidence of DKA in T1DM patients was 10.2%. From the recent data of young-onset (onset < 30 years of age) T1DM and T2DM patients at Siriraj Hospital, Thailand’s largest national tertiary referral center, 66.1% of T1DM patients and 13.7% of T2DM patients had DKA at presentation [17]. Therefore, newly diagnosed diabetic patients who presented with DKA as the first manifestation could have either T1DM or T2DM. After the resolution of DKA on the first day, all patients with either type of diabetes need insulin therapy for glycemic control due to beta cell dysfunction from glucose toxicity [18]. After several weeks to months, some T2DM patients can stop insulin therapy [15]. However, T1DM patients must use insulin lifelong due to permanent beta cell damage.

There have been few studies in Thailand on initially diagnosed diabetic patients who presented with DKA [19]. T1DM and T2DM are the most common types of diabetes with DKA [20]. Therefore, we conducted this study to classify the clinical characteristics and treatment outcomes of initially diagnosed diabetic patients who presented with DKA to help categorize the correct type of diabetes. Furthermore, this study aimed to identify the risk factors associated with mortality from DKA.

## Materials and methods

### Participants and study design

We conducted a retrospective cohort study in patients with DKA from 1 January 2005 to 31 December 2019 at Siriraj Hospital, Thailand’s largest tertiary hospital. Patients older than 18 years and who were initially diagnosed with diabetes mellitus presenting with DKA were included in the study. The diagnosis of DKA was made by clinical examination and laboratory investigations following the diagnostic criteria of the American Diabetes Association (ADA) in 2009 [21]: plasma glucose greater than 13.9 mmol/L, detected plasma or urinary ketone level, and wide anion gap metabolic acidosis (bicarbonate less than or equal to 18 mmol/L or anion gap greater than 10–12 mmol/L). We also classified the severity of DKA according to the ADA in 2009 [21]. Patients with gestational diabetes or pregnant or breastfeeding women were excluded from the study. To classify the type of diabetes, we collected data during hospital admission and within the 12-month follow-up period from medical records from Siriraj Hospital. The study protocol was approved by the Siriraj Institutional Review Board, Faculty of Medicine Siriraj Hospital, Mahidol University, Bangkok, Thailand.

### Data collection and definitions

We obtained clinical data, including demographic data, comorbidities, family history of diabetes mellitus, precipitating causes of DKA, body weight with calculated body mass index (BMI), and acanthosis nigricans, which is a sign of insulin resistance.

Laboratory investigations included plasma glucose using the enzymatic method (hexokinase), renal function (creatinine and estimated glomerular filtration rate (eGFR) using the Chronic Kidney Disease Epidemiology Collaboration (CKD-EPI) equation [22], and serum electrolyte using methods with indirect ion-selective electrode and enzymatic kinetic ultraviolet assay. Beta-hydroxybutyrate, one of the forms of ketone bodies, was measured as ketone in plasma with an enzymatic (beta-hydroxybutyrate dehydrogenase and diaphorase enzymes) technique (reference range 0.02–0.27 mmol/L). If arterial or venous blood gas tests were performed, the pH was collected to consider with serum bicarbonate to categorize the severity of the DKA (mild, moderate, and severe). We calculated the effective serum osmolarity to clarify the overlap of DKA and the hyperglycemic hyperosmolar state (HHS). Pancreatic autoantibodies (anti-GAD, anti-IA2, and anti-zinc transporter 8 (anti-ZnT8)) were measured with an enzyme-linked immunosorbent assay (reference range; positive > 1 U/mL).

The severity of DKA was classified as mild, moderate, or severe based on the following criteria [21]. Mild DKA was diagnosed if the patient had arterial pH 7.25–7.3, serum bicarbonate 15–18 mmol/L, or anion gap > 10. Moderate DKA was diagnosed if the patient had arterial pH 7.0 to < 7.24, serum bicarbonate 10 to < 15 mmol/L, or anion gap > 12. Severe DKA was diagnosed if the patient had arterial pH < 7.0, serum bicarbonate < 10 mmol/L, or anion gap > 12.

The definition of the types of diabetes was based on the criteria in the ADA Standards of Medical Care in Diabetes in 2020 [23] and the WHO classification of diabetes mellitus in 2019 [5]. We defined the types of diabetes according to the biochemical and clinical characteristics of the patients. Type 1 diabetes was diagnosed with positive pancreatic autoantibodies or fasting c-peptide tests less than 0.08 nmol/L [24]. If the patients had negative pancreatic autoantibodies or did not measure pancreatic autoantibodies and fasting C-peptide, the diagnosis was made by clinical criteria if the patients had normal to low body mass index (BMI less than 23 kg/m^2^) and could not discontinue insulin therapy within 12 months after the initial diagnosis of DKA. Type 2 diabetes was diagnosed by clinical criteria if the patients were overweight or obese (BMI greater than 23 kg/m^2^), had a first-degree relative family history of type 2 diabetes, or had signs of insulin resistance (acanthosis nigricans). If the patients did not fulfill the clinical criteria for type 2 diabetes, the diagnosis was made if the patients had a negative test of pancreatic autoantibodies and did not fulfill the conditions for type 1 diabetes and the other specific types of diabetes. Unclassified diabetes was diagnosed if the patients did not have complete data at admission or were lost to follow-up after discharge from the hospital.

### Study outcomes

The primary outcome of this study was to compare the clinical characteristics of initially diagnosed patients with T1DM and T2DM who presented with DKA as the first manifestation. The secondary outcome of this study was to examine the mortality rate of DKA, identify clinical risk factors associated with DKA mortality, and explore the natural history of the disease and long-term antidiabetic medications within 12 months after recovery from DKA in patients with T1DM and T2DM.

### Statistical analysis

Demographic and clinical data are presented as numbers with percentages, means with standard deviations, or medians with interquartile ranges. The chi-square test or Fisher’s exact test was used to compare categorical variables. Student’s *t*-test was used to compare continuous variables with normally distributed data. The nonparametric Mann–Whitney U test was used to compare continuous variables with skewed distributions. Binary logistic regression was used to analyze factors associated with mortality. All probabilities were two-tailed, and a *p* value of less than 0.05 was considered to indicate statistical significance. All statistical analyses were performed using SPSS Statistics, Version 28 (IBM SPSS, Armonk, NY, USA).

## Results

**Fig. 1:**
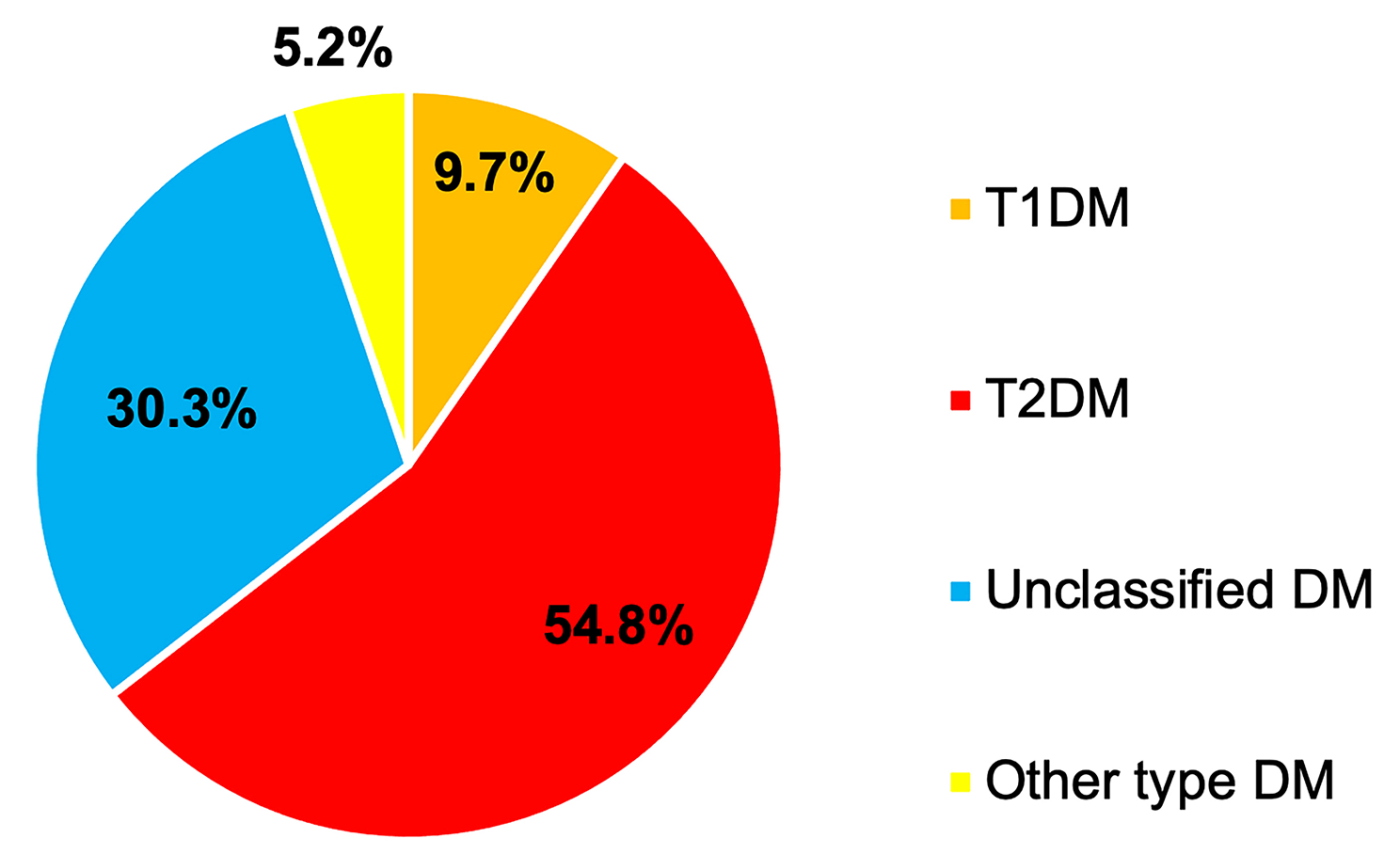
Classified type of diabetes in patients presenting with DKA at initial diagnosis (*N* = 155) Abbreviations: DKA: diabetic ketoacidosis; DM: diabetes mellitus; T1DM: type 1 diabetes mellitus; T2DM: type 2 diabetes mellitus

Five hundred thirty-nine patients with DKA were diagnosed between 1 January 2005 and 31 December 2019 at Siriraj Hospital. The prevalence of initially diagnosed diabetic patients who presented with DKA as the first manifestation was 28.8% (155 patients). The most common type of diabetes was T2DM (85 patients, 54.8%), and eight patients had other specific types of diabetes (5.2%), including steroid-induced diabetes and pancreatogenic diabetes (Fig. 1).

**Fig. 2:**
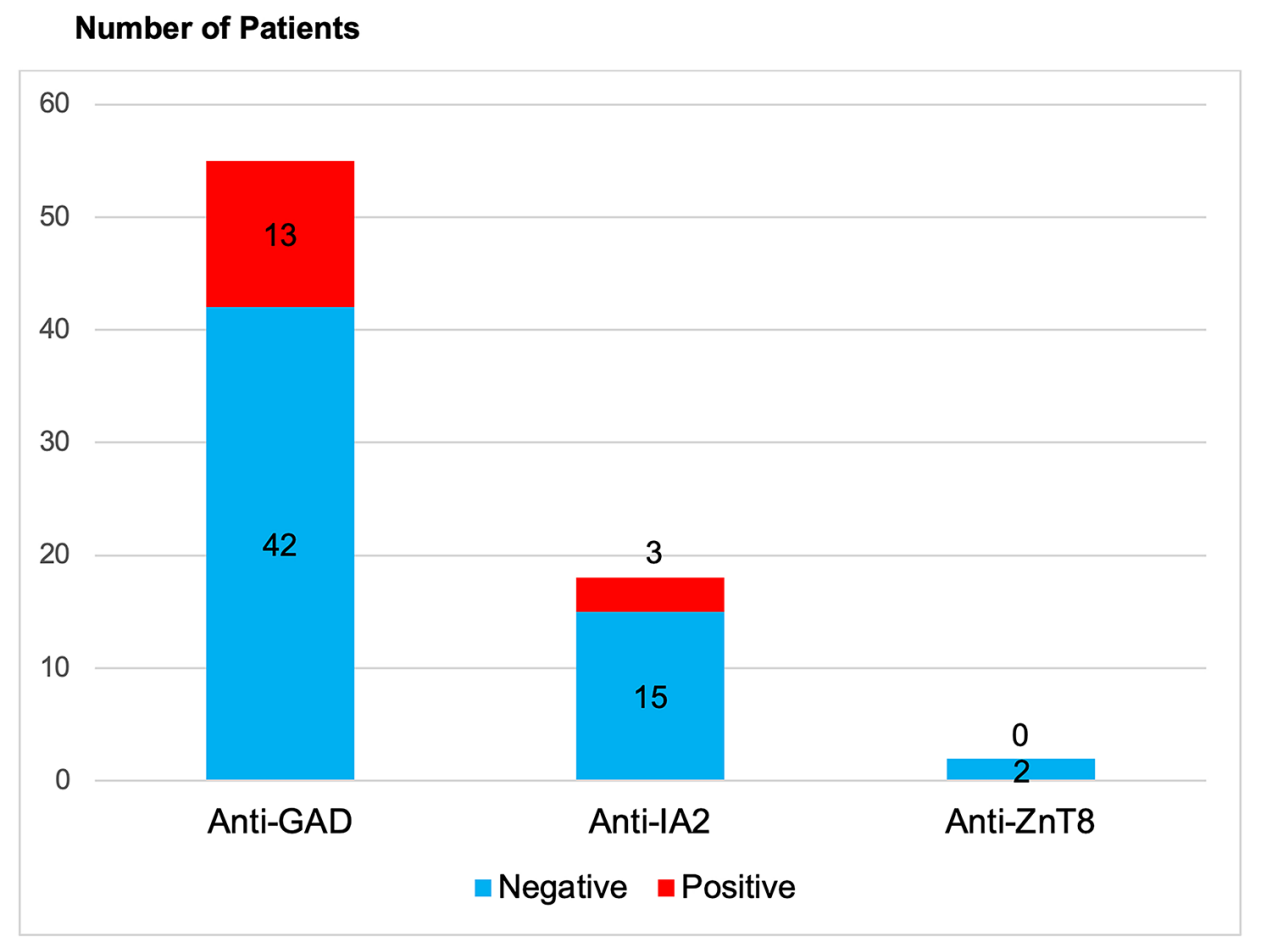
Type of pancreatic autoantibodies in initially diagnosed diabetic patients presenting with DKA (*N* = 75) Abbreviations: Anti-GAD: Anti-glutamic acid decarboxylase 65; Anti-IA2: Anti-islet antigen 2; Anti-ZnT8: anti-zinc transporter 8; DKA: diabetic ketoacidosis

Among the 155 patients (initially diagnosed diabetic patients who presented with DKA), a total of 100 patients (T1DM and T2DM) were analyzed (excluding unclassified diabetes and other specific types of diabetes). Pancreatic autoantibodies were tested in 55 patients. In Fig. 2, anti-GAD was positive in 23.6%, while only three patients were positive for anti-GAD and anti-IA2. None of the patients was positive for anti-ZnT8.

We examined the baseline characteristics of initially diagnosed patients with T1DM and T2DM presenting with DKA (Table 1). The male gender was predominant in both groups. Type 1 diabetes patients had a significantly lower mean age than T2DM patients (33 ± 16.2 vs. 51 ± 14.5 years, *p* value < 0.001). Type 2 diabetes patients had hypertension more than T1DM patients (21.2% vs. 6.7%), but the difference was not statistically significant (*p* value 0.291). A family history of diabetes (especially in first-degree relatives) was more common in T2DM patients than in T1DM patients (54% vs. 21.4%, *p* value 0.028). Type 1 diabetes patients had a significantly lower calculated BMI than T2DM patients (20.8 ± 4.6 vs. 26.4 ± 5.1 kg/m^2^, *p* value 0.001).

**Table 1:** Baseline characteristics of the initially diagnosed T1DM and T2DM patients presenting with DKA

	All Diabetes (*N* = 155)	T1DM (*N* = 15)	T2DM (*N* = 85)	*p* value*
Male	91 (58.7)	11 (73.3)	50 (58.8)	0.288
Body weight (kg)	66.3 *+ 17.3*	56.1 *±* 16.6	70.6 *±* 16.7	0.005
BMI (kg/m^2^)	25 *+ 5.5*	20.8 *±* 4.6	26.4 *±* 5.1	< 0.001
BMI < 21 kg/m^2^	22 (18.6)	7 (58.3)	6 (8)	< 0.001
BMI 21–24.9 kg/m^2^	47 (39.8)	3 (25)	31 (41.3)	0.352
BMI 25–30 kg/m^2^	32 (27.1)	1 (8.3)	24 (32)	0.167
BMI > 30 kg/m^2^	17 (14.4)	1 (8.3)	14 (18.7)	0.683
Systolic blood pressure^a^ (mmHg)	126 *+ 22.2*	134 *±* 23.8	127 *±* 24.4	0.328
Diastolic blood pressure^a^ (mmHg)	129 *+ 12*	75 *±* 14.5	79 *±* 16.9	0.417
Blood pressure *≥* 140/90 mmHg^a^	35 (22.6)	3 (20)	24 (28.2)	0.753
Presence of acanthosis nigricans	18 (29.5)	1 (6.7)	16 (42.1)	0.074
Mean age (years)	47 *+ 15.6*	33 *±* 16.2	51 *±* 14.5	< 0.001
**Age group**				
18–44 years	74 (47.7)	13 (86.7)	30 (35.3)	< 0.001
45–64 years	57 (36.8)	0	39 (45.9)	< 0.001
65–84 years	24 (15.5)	2 (13.3)	16 (18.8)	1.000
**Smoking status**				
Non-smoker	96 (68.6)	11 (73.3)	54 (68.4)	1.000
Current smoker	27 (19.3)	3 (20)	12 (15.2)	0.702
Ex-smoker	17 (12.1)	1 (6.7)	13 (16.4)	0.456
**Comorbidities**				
Hypertension	25 (16.1)	1 (6.7)	18 (21.2)	0.291
Dyslipidemia	14 (9)	0	11 (12.9)	0.209
Coronary artery disease	1 (0.6)	0	1 (1.2)	1.000
Cerebrovascular disease	4 (2.6)	0	2 (2.4)	1.000
Family history of DM	56 (49.1)	3 (21.4)	34 (54)	0.028

Notes: Data are reported as mean ± standard deviation or number (percentage), as appropriate. * *p*-value when T1DM was compared with T2DM. ^a^Blood pressure on the first day of DKA presentation

Abbreviations: BMI: body mass index; DKA: diabetic ketoacidosis; DM: diabetes mellitus; T1DM: type 1 diabetes mellitus; T2DM: type 2 diabetes mellitus

The precipitating factors of DKA were identified in 108 patients (69.7%). The most common cause was infection (55.5%), including respiratory tract infection and urinary tract infection (16.1% and 12.9%, respectively). In this study, we found some rare but life-threatening causes, such as thyroid storm or cerebral venous sinus thrombosis. Type 2 diabetes patients had more precipitating factors than patients with T1DM (76.5% vs. 33.3%, *p* value 0.002), as shown in Table 2.

**Table 2:** The precipitating factors of DKA in initially diagnosed T1DM and T2DM patients presenting with DKA

	T1DM (*N* = 15)	T2DM (*N* = 85)	*p* value
Presence of precipitating factors	5 (33.3)	65 (76.5)	0.002
Infection	5 (33.3)	50 (58.8)	0.067
Urinary tract infection	1 (6.7)	10 (11.8)	1.000
Respiratory tract infection	2 (13.3)	14 (16.5)	1.000
Gastrointestinal tract infection	1 (6.7)	5 (5.9)	1.000
Skin and soft tissue infection	0	8 (9.4)	0.602
Myocardial infarction	0	4 (4.7)	1.000
Glucocorticoid use	1 (6.7)	12 (14.1)	0.685
Herbal use	0	3 (3.5)	1.000
Pancreatitis	0	7 (8.2)	0.590

Note: Data are reported as number (percentage)

Abbreviations: DKA: diabetic ketoacidosis; T1DM: type 1 diabetes mellitus; T2DM: type 2 diabetes mellitus

During DKA, laboratory investigations at admission showed that the mean plasma glucose was 39.6 ± 18.5 mmol/L, and the mean glycated hemoglobin (HbA1c) was 13.2 ± 3.3%. When comparing T1DM and T2DM patients (Table 3), the mean plasma glucose was lower in T1DM patients than in T2DM patients (31.7 ± 12.2 mmol/L vs. 42.9 ± 22.1 mmol/L, *p* value 0.001). There was no difference in HbA1c between the two groups. Type 2 diabetes patients had significantly lower renal function than those with T1DM (mean eGFR 71.9 ± 39.9 vs. 105.2 ± 37.1 ml/min/1.73m^2^, *p* value 0.025). There was no difference in DKA management between T1DM and T2DM patients (Supplementary Table 1).

**Table 3:** Laboratory investigations at admission of the initially diagnosed T1DM and T2DM patients presenting with DKA

	All Diabetes (*N* = 155)	T1DM (*N* = 15)	T2DM (*N* = 85)	*p* value*
Plasma glucose (mmol/L)	39.6 *+ 18.5*	31.7 *±* 12.2	42.9 *±* 22.1	0.001
HbA_1c_ (%)	13.2 *+ 3.3*	12.7 *±* 4.7	13.6 *±* 3	0.483
eGFR (ml/min/1.73m^2^)	75.9 *+ 39.9*	105.2 *±* 37.1	71.9 *±* 39.9	0.025
Sodium (mmol/L)	129 *+ 9.4*	131 *±* 10.5	130 *±* 10.4	0.665
Potassium (mmol/L)	4.7 *+ 1*	4.6 *±* 1	4.8 *±* 1.1	0.536
Chloride (mmol/L)	89 *+ 9.8*	93 *±* 9.2	90 *±* 10.7	0.286
Bicarbonate (mmol/L)	10 *+ 5.3*	10 *±* 4.7	10 *±* 4.9	0.876
pH in arterial/venous blood gas	7.23 *+ 0.16*	7.21 *±* 0.14	7.25 *±* 0.14	0.323
Ketone (mmol/L)	5.1 *+ 2.3*	5.5 *±* 2.2	5.3 *±* 2.3	0.872
Effective serum osmolality (mOsmol/kg)	296 *+ 24.1*	293 *±* 23.1	300 *±* 26.8	0.331
Positive Anti-GAD^a^	13 (23.6)	13 (92.9)	0	< 0.001
Anti-GAD level (U/mL)^b^	0.07 (0.01–1596.9)	8.17 (3.63–24.66)	0.02 (0.01–0.11)	< 0.001
Positive Anti-IA2^a^	3 (16.7)	3 (42.9)	0	0.063

Notes: Data are reported as mean ± standard deviation or number (percentage), as appropriate. * *p*-value when T1DM was compared with T2DM. ^a^Total population who was tested for pancreatic autoantibodies, *N* = 55. ^b^Data are reported as median (25th -75th percentile)

Abbreviations: Anti-GAD: Anti-glutamic acid decarboxylase 65; Anti-IA2: Anti-islet antigen 2; DKA: diabetic ketoacidosis; eGFR: estimated glomerular filtration rate; HbA_1c_: glycated hemoglobin; T1DM: type 1 diabetes mellitus; T2DM: type 2 diabetes mellitus

The clinical outcomes of the initially diagnosed T1DM and T2DM patients with the first manifestation of DKA are shown in Table 4. More participants were admitted to the general ward than to the intensive care unit (ICU) (89.4% vs. 10.6%); fourteen patients were referred to the affiliated hospitals. Data were available in 138 patients for calculation of the DKA resolution time; 76.1% (105 of 138 patients) had DKA resolution within 24 h. Most T1DM patients had a moderate to severe form of DKA (13/15 patients, 86.7%), similar to T2DM patients (66/85, 78.6%). The median length of stay of T2DM patients was 8 days (interquartile range 6–12 days), which was significantly longer than that of T1DM patients (*p* value 0.023). Four type 2 diabetes patients (4%) died, but none with T1DM. The most common cause of death was septic shock and multiorgan failure. For other complications, there was more acute kidney injury in T2DM patients than in T1DM patients, but the difference was not statistically significant. The mean age of the death group was higher than that of the survival group, but without statistical significance (62 ± 12.2 vs. 47.9 ± 16, *p* value 0.101) (Supplementary Table 2). All patients in the death group had infections as the precipitating factors. The highest mortality was associated with moderate DKA (75%), but no statistical significance.

**Table 4:** The clinical outcomes of the initially diagnosed T1DM and T2DM patients presenting with DKA

	T1DM (*N* = 15)	T2DM (*N* = 85)	*p* value
**Severity of DKA**			
Mild	2 (13.3)	18 (21.4)	0.729
Moderate	7 (46.7)	31 (36.9)	0.474
Severe	6 (40)	35 (41.7)	0.904
**Admission**			
In general ward	14 (93.3)	71 (86.6)	0.457
In ICU	1 (6.7)	11 (13.4)	0.687
Length of stay (days)	6 (4–8)	8 (6–12)	0.023
Resolution time (hours)	17 (9–23)	15 (10–27)	0.628
Resolution time within 24 hours^a^	13 (86.7)	59 (72.8)	0.343
Death	0	4 (4.8)	1.000
Acute kidney injury^b^	4 (26.7)	40 (47.1)	0.133
Combined with HHS^c^	1 (6.7)	16 (19.3)	0.457

Notes: Data are reported as number (percentage) or median (25th -75th percentile), as appropriate. ^a^Data of resolution time within 24 h, *N* = 138. ^b^Data of acute kidney injury, *N* = 154. ^c^Data of combined with HHS, *N* = 153

Abbreviations: DKA: diabetic ketoacidosis; HHS: hyperglycemic hyperosmolar state; ICU: intensive care unit; T1DM: type 1 diabetes mellitus; T2DM: type 2 diabetes mellitus

After recovery from DKA and other medical illnesses, 85 of 146 patients (58.2%) were followed up at Siriraj Hospital. 67% of T2DM patients were followed up for 12 months. An increasing insulin discontinuation rate was observed every three months during the follow-up period. Within the 12-month follow-up period (Supplementary Table 3), there were no differences in body weight or total daily doses of insulin between the two groups, but T2DM patients achieved better glycemic control than T1DM patients. Over half of the T2DM patients (33 of 57, 57.9%) could discontinue insulin therapy.

## Discussion

In this retrospective study of 155 patients with initially diagnosed diabetes presenting with DKA, we found that more than half of the patients had T2DM and were male. Patients with T2DM were older, had a higher body mass index, more family history of diabetes, more precipitating factors, higher plasma glucose, and lower renal function than those with T1DM. There was no difference in DKA resolution time or management between T1DM and T2DM patients. Type 2 diabetes patients had more prolonged hospital admissions than T1DM patients.

Regarding the prevalence of newly diagnosed diabetic patients who presented with DKA as the first manifestation, Balasubramanyam et al. reported that 28% of patients who presented with DKA at Ben Taub General Hospital in the USA had new-onset diabetes [25], which was similar to our study. Another study in Thailand conducted between 2006 and 2010 reported that 21.7% of patients with hyperglycemic crises had newly diagnosed diabetes [26]. Our study conducted between 2005 and 2019 found that 28.8% of patients with DKA had newly diagnosed diabetes, which was higher than the previous study. This could be explained by the increased prevalence of diabetes mellitus in the Thai population during a 10-year period [3].

Some previous studies showed that most DKA episodes occurred in patients with T1DM [27–32]. However, our study showed that 54.8% and 9.7% of newly diagnosed diabetic patients who presented with DKA were classified with T2DM and T1DM, respectively. Some previous studies in the Asian population also showed that the majority of DKA patients were diagnosed with T2DM [20, 33–36]. The reason behind this finding may be due to the very low incidence of T1DM in Thailand (the crude incidence rate of 5/100,000 in 2015) compared to Europe and North America (the incidence rate of 12 to 32/10,000 in 2021) [37, 38]. In our study, there was a difference in clinical characteristics between the two types. T2DM patients were older, had greater body weight, and were more likely to have a family history of diabetes than T1DM patients. This was consistent with a previous study of Thai diabetic patients diagnosed with DKA [20]. According to various studies [19, 20, 26, 34], most T2DM patients had precipitating causes of DKA, especially infections, consistent with our study. On the other hand, previous studies [20, 34, 39] showed that nonadherence to treatment was the main precipitating factor of DKA in T1DM patients. Since our study was conducted in initially diagnosed diabetic patients, two-thirds of T1DM patients did not have a precipitating factor.

In our study, most of the patients were male, similar to previous studies in Thailand and Korea [19, 40]. T2DM patients had higher plasma glucose levels than T1DM patients, which may be explained by the insidious clinical onset of T2DM [4]. Furthermore, T2DM patients had a lower eGFR (using the CKD-EPI equation [22] than those with T1DM, possibly due to the older age of patients with T2DM. No differences were found in serum electrolytes, serum ketones, or pH levels. Some previous studies [20, 25] also found no difference in serum bicarbonate and ketone levels. However, another study [34] found lower serum bicarbonate and pH levels in T1DM patients than in T2DM patients.

Currently, detecting pancreatic islet autoantibodies is helpful in diagnosing T1DM [7]. In our study, the most prevalent islet autoantibody was anti-GAD, consistent with a previous study [41]. However, a recent systematic review in children and adolescents showed that anti-IA2 was the most prevalent islet antibody in new-onset T1DM globally [42]. However, the absence of autoantibodies does not exclude T1DM since approximately 15% of patients with the T1DM clinical phenotype have negative results [43]. Our study showed that 7% of T1DM patients were autoantibody negative.

There were no significant differences in the severity and resolution time of DKA between the two types of diabetes, which was consistent with previous studies [20, 44]. T1DM patients had a shorter length of stay than T2DM patients, similar to previous studies [20, 32, 45]. Because most T1DM patients were young and there were no precipitating factors requiring treatment, such as antibiotics, there was no need for more extended hospitalization.

In our study, the mortality rate of DKA was 4%. According to a previous study in Taiwan during 1992–1997 [33], the mortality rate of DKA in new-onset diabetes was 6.25%, similar to our study. Another study in Israel during 2003–2010 showed that the in-hospital mortality rate of DKA patients was 4.5% [30]. However, another study in Thailand during 2018–2020 showed no mortality in DKA patients [34]. The mortality rate has decreased over time because there is a large amount of updated clinical or research data that improve the quality of treatment. Furthermore, there is more screening for diabetes in the general population [46], which can prevent diabetic complications, including hyperglycemic emergencies.

Within 12 months after the resolution of DKA, approximately half of the patients with T2DM could discontinue insulin therapy, which was more than the previous study in Europe (32.4%) [15]. If we can accurately differentiate a type of diabetes in the newly diagnosed diabetic patients who presented with DKA, the appropriate monitoring and management, including discontinuation of insulin, will be implemented. Therefore, the results of our study could facilitate this decision-making process. Unfortunately, our study did not include types of diabetes other than T1DM and T2DM. Future studies on these groups of patients could be beneficial.

The strengths of our research include comprehensive data on initially diagnosed diabetic patients who presented with DKA with respect to clinical characteristics, laboratory investigations, management, and outcomes because there were few studies on these patients in Thailand [19]. Furthermore, this study provided new information, such as medications after DKA resolution for one year of initially diagnosed T2DM patients and clinical characteristics comparing T1DM and T2DM patients during the follow-up period.

Our limitations were as follows: first, some laboratory parameters, such as c-peptide levels or pancreatic autoantibodies, were unavailable in some patients because these investigations were not performed routinely at our institute. As a result, it hampers the chance of those with latent autoimmune diabetes of the adult (LADA), who could well masquerade as cases of T2DM for many years. Second, the results could not be applicable to younger patients or patients with other types of diabetes because our study only had detailed data on adult patients with T1DM and T2DM. Lastly, this study was retrospective and was done exclusively on the Thai population. Therefore, the results of the study could not be generalized.

## Conclusion

Our study contributes to the knowledge of DKA in initially diagnosed diabetic patients in Thailand. Adult patients who presented with DKA as the first manifestation were not always T1DM patients. Older age, obesity, a family history of diabetes, and precipitating factors were strong predictors of T2DM. T2DM patients had higher blood sugar at the initial manifestation of DKA, but there were no differences in HbA1c or other laboratory tests. More than half of the T2DM patients were able to stop insulin therapy within one year. If the correct type of diabetes is diagnosed, then physicians can provide proper management, leading to the discontinuation of insulin treatment with confidence and safety.

### Electronic supplementary material

Below is the link to the electronic supplementary material.

Supplementary Material 1

## Data Availability

The datasets used and analyzed during the current study are fully available from the corresponding author upon reasonable request.

## Abbreviations

ADA: American Diabetes Association
Anti-GAD: Anti-glutamic acid decarboxylase 65
Anti-IA2: Anti-islet antigen 2
Anti-ZnT8: Anti-zinc transporter 8
BMI: Body mass index
CKD-EPI: Chronic Kidney Disease Epidemiology Collaboration
DKA: Diabetic ketoacidosis
DM: Diabetes mellitus
eGFR: Estimated glomerular filtration rate
HbA_1c_: Glycated hemoglobin
HHS: Hyperglycemic hyperosmolar state
ICU: Intensive care unit
SPSS: Statistical Package for the Social Sciences
T1DM: Type 1 diabetes mellitus
T2DM: Type 2 diabetes mellitus
WHO: World Health Organization
