# Supplementary material for: Comparison of clinical characteristics and treatment outcomes between initially diagnosed type 1 and type 2 diabetes mellitus patients presenting with diabetic ketoacidosis
Source: BMC Endocr Disord. 2024 Jul 15;24:114. doi: 10.1186/s12902-024-01649-7 (PMC11247844; doi:10.1186/s12902-024-01649-7)
Supplement: Supplementary file 1 — Supplementary Material 1 [file 12902_2024_1649_MOESM1_ESM.docx]

**Supplementary Table 1. The DKA treatment of the initially diagnosed diabetic patients presenting with DKA**

|  | **T1DM and T2DM**  **(N=100)** | **T1DM**  **(N=15)** | **T2DM**  **(N=85)** | ***p* value^*^** |
| --- | --- | --- | --- | --- |
| Total fluid volume resuscitation within 24 hours of admission (ml)^a^ | 4508  (3400-6495) | 3850  (3000-5960) | 4635  (3468-6775) | 0.361 |
| Treatment of potassium within 4 hours^b^ | 40 (42.1) | 6 (40) | 34 (42.5) | 0.857 |
| Route of insulin^c^ |  | | | |
| Intravenous route | 91 (94.8) | 15 (100) | 76 (93.8) | 0.586 |
| Intramuscular route | 5 (5.2) | 0 | 5 (6.2) | 1.000 |
| Home medication^d^ |  | | | |
| Insulin use only | 68 (73.1) | 15 (100) | 54 (68.4) | 0.010 |
| Insulin plus oral antidiabetic drugs | 16 (17.2) | 0 | 16 (20.3) | 0.118 |
| Oral antidiabetic drugs use only | 9 (9.7) | 0 | 9 (11.4) | 0.346 |
| Insulin regimen^e^ |  | | | |
| Basal insulin regimen | 4 (4.8) | 0 | 4 (5.7) | 1.000 |
| Premixed insulin regimen | 72 (85.7) | 9 (60) | 63 (90) | 0.009 |
| Basal-bolus regimen | 8 (9.5) | 6 (40) | 3 (4.3) | <0.001 |
| Total daily doses of insulin (units/day)^a^ | 47 (30-59) | 49 (30-54) | 46 (30-60) | 0.810 |

**Notes:** Data are reported as mean ± standard deviation or number (percentage), as appropriate. ^*^*p*-value when T1DM was compared with T2DM. ^a^Data are reported as median (25^th^-75^th^ percentile). ^b^Data of treatment of potassium within 4 hours, N=147. ^c^4 data were missing. ^d^7 data were missing. ^e^Data of insulin regimen, N=84.

**Abbreviations:** DKA, diabetic ketoacidosis; T1DM, type 1 diabetes mellitus; T2DM, type 2 diabetes mellitus.

**Supplementary Table 2. Characteristics of initially diagnosed diabetic patients presenting with DKA compared between death and survival groups**

|  | **Survival**  **(N=96)** | **Death**  **(N=4)** | ***p* value** |
| --- | --- | --- | --- |
| Male | 57 (59.4) | 4 (100) | 0.154 |
| Age (years) | 47.9 + 16 | 62 + 12.2 | 0.101 |
| BMI (kg/m^2^) | 25.7 + 5.4 | 23.1 + 2 | 0.132 |
| Precipitating factor | 66 (68.8) | 4 (100) | 0.313 |
| Infection | 51 (53.1) | 4 (100) | 0.125 |
| Plasma glucose (mmol/L) | 41.6 + 21.1 | 27.1 + 23.5 | 0.396 |
| HbA_1c_ (%) | 13.4 + 3.3 | 14.4 + 3.7 | 0.652 |
| ICU admission | 10 (10.8) | 2 (50) | 0.074 |
| Acute kidney injury | 42 (44.2) | 2 (50) | 1.000 |
| Combined with HHS | 17 (17.9) | 0 | 1.000 |
| **Severity of DKA** |  |  |  |
| Mild  Moderate  Severe | 20 (21.1)  35 (36.8)  40 (42.1) | 0 (0)  3 (75)  1 (25) | 0.580  0.156  0.640 |

**Note:** Data are reported as mean ± standard deviation or number (percentage), as appropriate.

**Abbreviations:** BMI, body mass index; DKA, diabetic ketoacidosis; HbA_1c_, glycated hemoglobin; HHS, hyperglycemic hyperosmolar state; ICU, intensive care unit; T1DM, type 1 diabetes mellitus; T2DM, type 2 diabetes mellitus.

**Supplementary Table 3. Characteristics of the initially diagnosed T1DM and T2DM patients presenting with DKA during follow-up**

|  | **All diabetes**  **(N=155)** | **T1DM**  **(N=15)** | **T2DM**  **(N=85)** | ***p* value^*^** |
| --- | --- | --- | --- | --- |
| **Follow-up within 3 months** | 84 (57.5)  (N=146) | 13 (86.7)  (N=15) | 56 (69.1)  (N=81) | 0.220 |
| body weight (kg) | 67.4 + 17 | 65 + 16.6 | 71 + 16.9 | 0.257 |
| Fasting plasma glucose (mmol/L) | 7.4 + 4.3 | 8.6 + 3.8 | 7.4 + 3.9 | 0.321 |
| HbA_1c_ (%) | 7.8 + 1.9 | 8.7 + 2.7 | 7.7 + 1.6 | 0.338 |
| Total daily doses of insulin (units/day) | 39 + 18 | 39 + 20 | 41 + 18.4 | 0.772 |
| **Follow-up within 3-6 months** | 70 (47.9)  (N=146) | 11 (73.3)  (N=15) | 51 (63)  (N=81) | 0.440 |
| Body weight (kg) | 66.5 + 17 | 68 + 15.7 | 68 + 17 | 0.964 |
| Fasting plasma glucose (mmol/L) | 7.5 + 3.6 | 9.1 + 5.8 | 7.2 + 2.7 | 0.351 |
| HbA_1c_ (%) | 6.8 + 1.4 | 7.5 + 2.1 | 6.6 + 1.1 | 0.233 |
| Total daily doses of insulin (units/day) | 36 + 17.8 | 39 + 14.7 | 35 + 18.7 | 0.532 |
| **Follow-up within 6-9 months** | 60 (41.1)  (N=146) | 11 (73.3)  (N=15) | 43 (53.1)  (N=81) | 0.147 |
| Body weight (kg) | 67.8 + 18.7 | 66 + 12.9 | 69 + 19.6 | 0.583 |
| Fasting plasma glucose (mmol/L) | 7.7 + 3.3 | 8.2 + 4.2 | 7.7 + 3.3 | 0.673 |
| HbA_1c_ (%) | 7.0 + 1.6 | 7.9 + 2.8 | 6.8 + 1.1 | 0.228 |
| Total daily doses of insulin (units/day) | 39 + 24.4 | 38 + 19.7 | 40 + 27.3 | 0.882 |
| **Follow-up within 9-12 months** | 54 (34.8)  (N=146) | 9 (60)  (N=15) | 40 (49.4)  (N=81) | 0.450 |
| Body weight (kg) | 68.9 + 19 | 71 + 12.2 | 70 + 20.1 | 0.913 |
| Fasting plasma glucose (mmol/L) | 7.9 + 3.5 | 8.3 + 2.8 | 7.9 + 3.9 | 0.800 |
| HbA_1c_ (%) | 7.0 + 1.5 | 7.2 + 1.9 | 6.9 + 1.5 | 0.574 |
| Total daily doses of insulin (units/day) | 41 + 25.7 | 40 + 22.3 | 44 + 28.3 | 0.727 |
| Discontinuation of insulin^a^ | 38 (44.7) | 0 | 33 (57.9) | <0.001 |

**Notes:** Data are reported as mean ± standard deviation or number (percentage), as appropriate. ^*^*p* value when T1DM was compared with T2DM. ^a^Data of insulin discontinuation, N=85.

**Abbreviations:** DKA, diabetic ketoacidosis; HbA_1c_, glycated hemoglobin; T1DM, type 1 diabetes mellitus; T2DM, type 2 diabetes mellitus
